# VenomPred: A Machine Learning Based Platform for Molecular Toxicity Predictions

**DOI:** 10.3390/ijms23042105

**Published:** 2022-02-14

**Authors:** Salvatore Galati, Miriana Di Stefano, Elisa Martinelli, Marco Macchia, Adriano Martinelli, Giulio Poli, Tiziano Tuccinardi

**Affiliations:** 1 Department of Pharmacy, University of Pisa, 56126 Pisa, Italy; salvatore.galati@phd.unipi.it (S.G.); miriana.distefano@phd.unipi.it (M.D.S.); e.martinelli3@studenti.unipi.it (E.M.); marco.macchia@unipi.it (M.M.); marti@adrianomartinelli.it (A.M.); tiziano.tuccinardi@unipi.it (T.T.); 2 Department of Life Sciences, University of Siena, 53100 Siena, Italy; 3 Center for Biotechnology, Sbarro Institute for Cancer Research and Molecular Medicine, College of Science and Technology, Temple University, Philadelphia, PA 19122, USA

**Keywords:** in silico toxicity, machine learning, artificial intelligence, mutagenicity, carcinogenicity, hepatoxicity, estrogenicity

## Abstract

The use of in silico toxicity prediction methods plays an important role in the selection of lead compounds and in ADMET studies since in vitro and in vivo methods are often limited by ethics, time, budget and other resources. In this context, we present our new web tool VenomPred, a user-friendly platform for evaluating the potential mutagenic, hepatotoxic, carcinogenic and estrogenic effects of small molecules. VenomPred platform employs several in-house Machine Learning (ML) models developed with datasets derived from VEGA QSAR, a software that includes a comprehensive collection of different toxicity models and has been used as a reference for building and evaluating our ML models. The results showed that our models achieved equal or better performance than those obtained with the reference models included in VEGA QSAR. In order to improve the predictive performance of our platform, we adopted a consensus approach combining the results of different ML models, which was able to predict chemical toxicity better than the single models. This improved method was thus implemented in the VenomPred platform, a freely accessible webserver that takes the SMILES (Simplified Molecular-Input Line-Entry System) strings of the compounds as input and sends the prediction results providing a probability score about their potential toxicity.

## 1. Introduction

The use of artificial intelligence (AI) and, in particular, machine learning (ML) in toxicology is increasingly widespread due to the reduced costs and time required by in silico approaches compared to in vivo and in vitro studies, the ethical concerns related to animal experiments and the possibility to handle and process a large amount of data through ML models. With the need to harmonize toxicology data and spread the knowledge of their results, new protocols have been implemented and shared among public and private companies. Back in 2006, the European Union completely revised its regulatory framework for chemicals, issuing new regulations on Registration, Evaluation, Authorization and Restriction of Chemicals (REACH) [1]. The idea behind the REACH protocol is to regulate the production and use of chemicals, as well as to assess and control their potential impact on humans and the environment, highly sustaining the application of high-throughput in silico toxicity predictions to reduce animal testing and research costs. In order to implement this regulation, the European Chemicals Agency (ECHA) [2] was created. The agency issued the ECHA Guidance on information requirements and chemical safety assessment, which gives in-depth background details and recommendations addressing the use of non-testing methods on chemicals. Recently, the eTOX project, active between 2010 and 2016, developed by the European Innovative Medicines Initiative (IMI) [3], aimed at the creation of a database collecting all safety data gathered from both pharmaceutical and public toxicology reports, together with the in silico prediction of the toxicological profiles of small molecules in early stages of drug discovery [4]. Finally, in the United States, the “Toxicology in the 21st Century” (TOX21) [5] was created, providing a long-term strategy aimed at reducing animal testing and enhancing the use of computational toxicology and bioinformatics. Among computational approaches, web tools are becoming more and more widely employed due to their free availability and the fact that no expert computer skills are necessary to use them [6]. Thus, the development of web tools for toxicity prediction has a great impact in reducing time and resources in drug discovery. Due to the necessity to easily and quickly evaluate and share the toxicity profile of small molecules, different open-source platforms were created by collaborations of both academic and industrial groups. Among these, VEGAHub [7] is one of the most complete free online platforms in terms of reported toxicity models and benchmark datasets. VEGAHub also provides the freely available software VEGA QSAR, able to perform toxicity predictions employing in silico methods and models reported in the literature and implemented therein. Most of the models report information about the training set of compounds used for their development, together with the corresponding results obtained during reliability assessment, and often provide the actual dataset of compounds available for download. Moreover, information about the test set of compounds used for the external evaluation of the models may also be provided, together with data related to the obtained performance and the actual set of molecules to be downloaded.

In this work, new ML models for toxicology predictions, addressing four different toxicity endpoints, were developed based on the datasets obtained from selected models included in VEGA QSAR. In particular, we focused on the prediction of mutagenic, carcinogenic, hepatotoxic and estrogenic effects of small molecules. In fact, mutagenicity and carcinogenicity are the most common short- and long-term consequences of prolonged exposure to substances, and they are among the most lethal and studied side effects. Moreover, hepatotoxicity and estrogenic activity were considered since hepatic cells and metabolic enzymes, as well as estrogen receptors, are most often subjected to interaction with xenobiotics, and they are one of the main points of the European “Green Deal” discussion that has set the goal of reducing environmental exposure of endocrine disruptors. After developing multiple models focused on the four different endpoints, in order to evaluate the possibility of improving their predictive performance, a consensus approach combining the results of their predictions was applied. As this approach was able to predict chemical toxicity better than the single models, it was thus implemented in the VenomPred platform, a freely accessible web tool that takes the SMILES strings of the compounds as an input, providing a probability score about their potential toxicity as an output.

## 2. Materials and Methods

### 2.1. Modeling Datasets

The compounds datasets were collected from models available within the VEGA QSAR platform. With the aim of developing binary classification models for toxicity predictions, we selected datasets of compounds employed in binary classification models included in VEGA QSAR, for which both training and test sets were available for download. These datasets were then used to train and test our models, respectively. The datasets for building our mutagenicity models were chosen from the model elaborated from the “CAESAR Workshop on QSAR Models for REACH” [8], which provides a qualitative prediction of mutagenicity based on the experimental Ames test [9,10] by combining two different methods. The first, which is reported as Model A, consists of a trained Support Vector Machine (SVM) classifier that labels molecules as either mutagenic or non-mutagenic. The second model, described as Model B, screens the non-mutagenic compounds against reportedly known structural alerts (SAs) to check if they have been correctly predicted as harmless. The datasets for the carcinogenicity endpoint were extracted from the “Carcinogenicity CAESAR” model” [11], based on a Counter Propagation Artificial Neural Network (CP-ANN) algorithm. The estrogenicity models were developed using the datasets extracted from the “Estrogen Receptor Relative Binding affinity model (IRFMN)” [12], based on a classification and regression tree (CART) algorithm. Hepatotoxicity models were built employing datasets derived from a binary hepatotoxicity model based on the SarPy software and molecules with known experimental activity, collected in a collaboration between VEGA QSAR developers and IRFMN [13]. It is worth mentioning that the reference mutagenicity and hepatotoxicity models included in VEGA QSAR were unable to provide a proper binary prediction for some of the compounds included in the test set employed for their validation, classifying them as “suspected” toxic. In fact, as reported in the corresponding documentation available in VEGA QSAR for these two models, the statistical performance related to the prediction of the test set was evaluated, omitting these compounds. Therefore, in order to properly compare the performance of our models with the reference ones included in VEGA QSAR, the same compounds were removed from the original test set of the mutagenicity and hepatotoxicity models prior to the statistical evaluation. The composition of each dataset employed for building and evaluating all developed ML models, including those implemented in the VenomPred platform, is reported in Table 1.

### 2.2. Molecular Fingerprints

The SMILES (Simplified Molecular-Input Line-Entry System) strings of training and test set compounds used for developing and testing our ML models for binary classification were downloaded from VEGA QSAR software. The SMILES strings were used to compute five different types of molecular fingerprints (FPs) [14], which were used as input for all developed ML models, including those implemented in VenomPred. Two open-source python libraries were used to compute molecular FPs: RDKit [15] and PyFingerprint [16]. The former was used to calculate Morgan, RDKit and Pharm2D FPs, while the second to compute PubChem and LINGO FPs.

*Morgan FPs* represent the atoms of the compounds based on the neighboring atoms and bonds within a determined atom distance (which was set to 2 in this work) and assign them a unique identifier. These identifiers are then usually hashed to a bit vector with a fixed length in order to allow the comparison of different representations. In this work, we set a vector length of 1024 bits using the Morgan FPs implementation of RDKit [15] since it was found that the use of a larger vector size with ML algorithms such as random forest could only allow negligible performance improvements [17].

*RDKit FPs* are RDKit-specific fingerprints inspired by public descriptions of the daylight topological fingerprints. The fingerprinting algorithm identifies all subgraphs in the molecule within a particular range of sizes, hashes each subgraph to generate a raw bit ID, adjust the raw bit ID to fit in the assigned fingerprint size and then sets the corresponding bit. In this work, a vector size of 1024 bits was used for RDKit FPs for consistency with Morgan and LINGO FPs.

*Pharm2D FPs* are 2D pharmacophore fingerprints created combining a set of chemical features with their 2D (topological) distances. When the distances are binned, unique integer values can be assigned to each of these pharmacophores and stored into a fingerprint. These fingerprints are calculated using a signature factory, which keeps track of all the parameters required to generate the pharmacophore, a task that is performed using the corresponding RDKit module (*rdkit.Chem.Pharm2D.Generate*). The pharmacophore fingerprints were computed considering all possible combinations of default features included within the RDKit library. Specifically, the following feature types were considered: H-bond acceptor, H-bond donor, positive ionizable, negative ionizable and aromatic features. Each feature combination included a minimum of two and a maximum of three features.

*PubChem FPs* [18] belong to substructure-based FPs included in a vector of 881 bits, in which each bit represents the presence of an element or substructure, the count of a ring system, the atom pairs and the atom’s nearest neighbors.

*LINGO FPs* [19] are based on the fragmentation of SMILES strings into substrings. SMILES strings are the most compact text-based molecular representations that contain the information needed to compute all kinds of molecular substructures and to derive molecular properties. For a SMILES string of length L, a number N of substrings of length Q (where N = L − Q + 1) is extracted; the occurrences of the substrings are then counted to provide the final LINGO profile. In this work, the default vector size of 1024 bits was used for LINGO FPs.

### 2.3. Classification Models

Four different classification algorithms were used to build the predictive toxicology models developed and included in the VenomPred platform: Random Forest, Support Vector Machine, k-Nearest Neighbor and Multi-Layer Perceptron. The proper functions of the python library Scikit-learn [20] were used for the generation of the models.

*Random Forest (RF).* The RF algorithm consists of a large number of individual trees that operate as an ensemble. Each individual tree in the random forest determines a class prediction [21]. The class that obtains the majority of votes becomes the ultimate prediction of the model, following the “wisdom of crowds” approach. The main hyperparameters optimized during model building were *max_features*, which indicates the maximum number of features that can be considered in a single tree, and *n_estimators*, expressing the number of trees built before making the averages of predictions. The options of *max_features* investigated were: (a) *sqrt*, which is the square root of the total features in a single node; (b) *log2*, which corresponds to the binary logarithm of the total features for a single node; (c) *None*, for which *max_features* corresponds to the total number of features. The number of estimators that were taken into account corresponds to 100 and 500.

*Support Vector Machine (SVM).* SVM maps the data according to their common patterns and aims towards their optimal division between two classes, with each of them entirely lying on opposite sides of a separating hyperplane. The goal is reached by maximizing the distance between the closest training data points, the so-called support vectors and the hyperplane [22]. The hyperparameters optimized during model building were: (a) the *kernel*, which is a function used to map the data into higher dimensional feature space in order to make them separable; and (b) *C*, which indicates how much emphasis is made on the misclassified data, therefore helping in optimizing the hyperplane.

*k-Nearest Neighbor (KNN).* The KNN algorithm classifies instances on the basis of a majority vote of their neighbors. Each test instance is predicted to fall into the class identified by its k-closest neighbors. The final prediction is therefore obtained by the most frequent outcome among the features of the nearest neighbors to the input data [23]. The hyperparameters optimized during model building were those that reduce the error due to the voting of the surrounding neighbors [24], namely *n_neighbors* and *weight*. *n_neighbors* indicates the number of neighbors taken into consideration for the classification, and *weight* defines how much the different surrounding elements influence the prediction. The values investigated for *n_neighbors* were in a range between 1 and 30, while two options were tested for *weight*: (a) *uniform*, indicating that all points in each neighborhood are weighted equally, and (b) *distance*, imposing that closer neighbors of a query point have a greater influence than neighbors that are further away from it.

*Multi-Layer Perceptron (MLP).* MLP is a type of feedforward artificial neural network (ANN) composed of multiple layers of nodes, which uses a supervised learning technique called backpropagation [25]. Four hyperparameters were tuned in order to minimize the error in the path from the input to the output predictions: (a) *hidden_layer_size*, which determines the number of neurons and the number of hidden layers; (b) *solver*, which is fundamental to optimize the predictions at every decision step through the different layers; (c) *activation*, which refers to the activation function and defines how the weighted sum of the input is transformed into output by one or more nodes in a network layer; (d) *learning_rate_init*, which controls the step-size in updating the weights. For the *hidden_layer_size*, we evaluated all possible combinations of a set of 100, 200 and 1000 neurons. As the type of *solver,* we tested *lbfgs*, which uses a limited amount of computer memory, only storing a certain amount of vectors, as well as the stochastic gradients *adam* and *sgd*. Among the activation functions, we considered *“identity”*, *“logistic”*, *“tanh”* and *“relu”* functions. The options investigated for *learning_rate_init* were 0.01, 0.001 and 0.0001.

### 2.4. Model Building and Evaluation

As previously described, 5 different molecular FPs and 4 different ML algorithms were employed for generating our toxicity models. In particular, for each endpoint considered, each FP type was combined with each algorithm, thus obtaining 20 different toxicity models for each type of classification. An optimization process based on the Grid Search cross-validation implemented in Scikit-learn was applied to all generated models for determining the best hyperparameters setting. Grid Search is a generic approach provided in Scikit-learn for parameter search. It employs cross-validation to explore all possible combinations of hyperparameters, assigning a score to each of them. In this work, the scoring parameter used is Matthew’s Correction Coefficient (see next section for details). In order to evaluate the performance of the 20 optimized models thus obtained for each endpoint, further 20-fold cross-validation (CV) was performed for each model using a random training-test set splitting strategy, in which 30% of the starting dataset was considered as the test set. After ranking the 20 different models according to Matthew’s Correction Coefficient, the top 5 models were selected for further analysis.

### 2.5. Final Model Evaluation Metrics

In order to check the performance of the 5 top-scored models selected for each endpoint, five statistical parameters were taken into account: Precision, Specificity, Recall (or Sensitivity) and Matthew’s Correction Coefficient (MCC), which are defined as follows:

(1) $$\mathrm{Precision}\;=\;\frac{TP}{\left(TP+FP\right)}$$

(2) $$\mathrm{Specificity}\;=\;\frac{TN}{\left(TN+FP\right)}$$

(3) $$\mathrm{Recall}\;=\;\frac{TP}{\left(TP+FN\right)}$$

(4) $$\mathrm{MCC}\;=\;\frac{\left(TPx\;TN\;–\;FPxFN\right)}{\sqrt{\left(TP+FP\right)\left(TP+FN\right)\left(TN+FP\right)\left(TN+FN\right)}}$$

*TP* (true positives) and *TN* (true negatives) correspond to the number of toxic and non-toxic compounds, respectively, correctly predicted as such; *FP* (false positives) represents the number of non-toxic compounds predicted as toxic and *FN* (false negatives) represents the number of toxic compounds predicted as non-toxic. Therefore, Precision indicates the percentage of correct positive predictions, while Specificity and Recall measure the ability of models to correctly predict negative and positive instances, respectively. MCC takes into account all values derived from binary classification and represents a balanced evaluation of the classifier performance [26]. For instance, MCC = 1 indicates a perfect classification (with no *FP* and *FN*), MCC = 0 is equivalent to random classification and MCC = −1 indicates a complete disagreement between predicted and actual classes.

Within the documentation available for the reference models included in VEGA QSAR, the MCC values related to the test set predictions were not present. Therefore, we calculated the MCC values after performing the test set predictions and computing the number of *TP*, *FP*, *TN* and *FN* for each considered reference model.

### 2.6. Consensus Strategy and Consensus Score

The consensus strategy consists in combining the predictions produced for each tested molecule by the top-scored models of each endpoint, which constitutes the predictive approach employed by the VenomPred platform. Each ML model returns a probability score (PS) ranging from 0 to 1 associated with the toxicity prediction generated for each compound (0 ≤ PS < 0.5 if predicted as non-toxic; 0.5 ≤ PS ≤ 1 if predicted as toxic); in particular, the closer the PS to 1, the higher the prediction confidence of toxicity, while the closer the PS to 0, the higher the prediction confidence of non-toxicity. Therefore, a consensus score (CS) for each tested molecule is computed by averaging the PSs produced by the top-scored models. In particular, we calculated three different consensus scores, namely CS3, CS4 and CS5, by averaging the PSs of the 3, 4 and 5 top-scored models, respectively. Accordingly, by using the consensus strategy, a compound was labeled as non-toxic if the obtained CS was lower than 0.5 and toxic if the CS was equal to or greater than 0.5. The same statistical parameters used for evaluating the performance of the single top-scored models (Precision, Specificity, Sensitivity and MCC) were then calculated based on the three different CS classifications in order to assess the performance of the consensus strategy and identify the best approach.

## 3. Results and Discussion

### 3.1. Model Generation, Optimization and Selection

With the aim of developing ML models for predicting the potential toxicological effect of small molecules and comparing their performance with known toxicity models reported in the literature, we used VEGA QSAR software (hereafter referred to as VEGA) as a reference. VEGA is a stand-alone application that includes models for small molecule toxicity predictions. It allows the analysis of several toxicity endpoints providing in-depth details and supporting information, making it one of the most comprehensive computational platforms for this type of study. In particular, VEGA was employed as a source of validated benchmark datasets for training, developing and evaluating our ML models, as well as a source of reference predictive tools to which the performance of our models could be compared. The toxicity endpoints that were analyzed in this work are mutagenicity, carcinogenicity, estrogenicity and hepatotoxicity. The choice of analyzing mutagenicity and carcinogenicity endpoints derives from their great importance on the long-term side effects of new drugs. In fact, this is a fundamental aspect of verifying the safety of drugs and, more generally, of chemicals on the market. This type of toxicity is linked to compounds that can alter the genetic material within germ cells, increase the rate of human diseases of genetic origin in the population and generate potential carcinogenic effects. On the other hand, hepatotoxicity is a major challenge to the pharmaceutical industry and regulatory authorities [27]. It is often a reason for drug failure and consequently for withdrawal from the market after approval, despite the preclinical and clinical evaluation of the safety of drug candidates [28]. Finally, estrogenicity arising especially from aquatic pollution [29] is the ability of exogenous substances to interfere with the function of the endocrine system, including those related to developmental processes. The estrogenic activity of small molecules and chemicals represents a source of concern, which is rising more and more interest not only in the scientific community; indeed, constituting one of the key points of the European Green Deal proposed by the European Commission, and it is thus another important issue to be addressed in toxicity evaluations. For each of these four endpoints, we selected a suitable model among those included in VEGA software to be used as a reference. Each selected model was used both as a source of training and test set compounds for developing and evaluating our ML models, respectively, and as a reference for comparing their predictive performance. For each endpoint, we thus selected as a reference the corresponding binary classification model included in VEGA trained and validated with the highest number of compounds, for which both training and test set molecules were fully available together with the corresponding performance evaluation results.

Training and test sets used for generating our mutagenicity models were obtained from the CAESAR mutagenicity model [8], which provides a qualitative prediction of mutagenicity on Salmonella typhimurium according to the Ames test [9,10], while carcinogenicity data were collected from the “Carcinogenicity CAESAR” [11] model. The datasets for the remaining endpoints were retrieved from the reference “Estrogen Receptor Relative Binding Affinity model” [12] and “Hepatotoxicity model (IRFMN)” [13]. Detailed information about the composition of the data sets is reported in the Materials and Methods section (Table 1).

**Figure 1:**
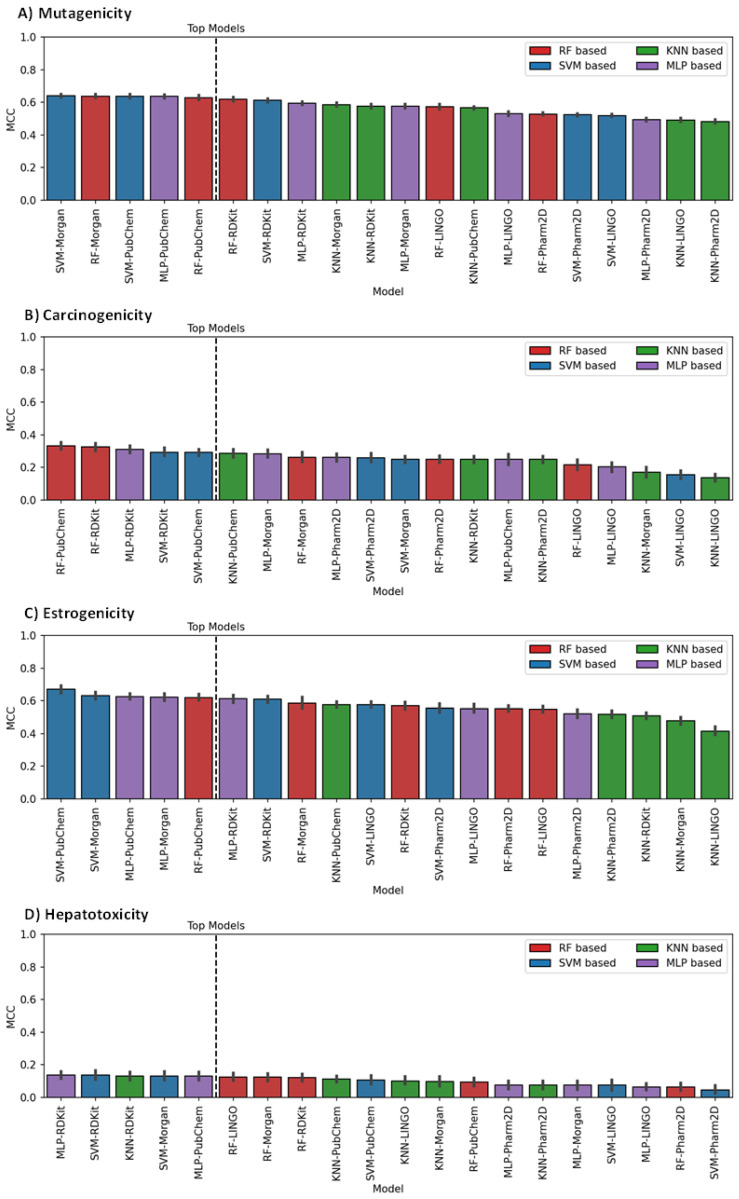
Evaluation results, expressed in terms of MCC, obtained for all different models developed for the (**A**) mutagenicity, (**B**) carcinogenicity, (**C**) estrogenicity and (**D**) hepatotoxicity endpoints. The 5 top-scored models for each endpoint are separated from the others by a black dashed line.

The generated models were obtained by representing the training set compounds through molecular fingerprints (FPs), which were generated from the SMILES notations of the compounds. In particular, five different types of FPs were used: Morgan, RDKit, Pharm2D, PubChem and LINGO FPs. Each FP method was combined with four different classification algorithms, i.e., Random Forest (RF), Support Vector Machine (SVM), k-Nearest Neighbor (KNN) and Multi-Layer Perceptron (MLP), for the development of 20 different classification models for each of the four different endpoints. After a hyperparameter optimization, the performance of all models of each endpoint was evaluated and ranked according to the values of Matthew’s Correction Coefficient (MCC) obtained through cross-validation (CV) analysis (see Materials and Methods for details). The results are summarized in Figure 1.

The results highlight that, overall, SVM appears to be the best algorithm since it is the only one included in two out of the five top-scored models for each endpoint. On the contrary, only one model based on the KNN algorithm is shown among the top-scored ones (for the hepatotoxicity endpoint). In terms of FPs, we observe the exclusive presence of Morgan, RDKit and, especially, PubChem FPs in the top-scored models, which thus demonstrate to provide a better molecular description for toxicological ML models with respect to Pharm2D and LINGO FPs. The performance reported in Figure 1 shows that for all endpoints, our ML models performed better than random predictions, as confirmed by the MCC scores above 0.

In particular, high performances were obtained for the mutagenicity and estrogenicity endpoints, for which almost all models achieved an MCC greater than 0.5 (Figure 1A,C); in fact, the average MCC of the five top-scored models corresponded to 0.63 in both cases. For the carcinogenicity endpoint, lower values of MCC (below 0.4) were generally obtained; interestingly, the RF algorithm showed to perform well for this type of prediction, since the two top-scored models, based on RF, were the only ones for which an MCC value higher than 0.35 was obtained (Figure 1B). On the contrary, no RF-based model appeared among the top-scored hepatotoxicity models, which showed generally poor performance, with MCC values below 0.2 (Figure 1D). Notably, carcinogenicity and hepatotoxicity were the only endpoints for which RDKit FPs were found among the best combinations of ML models.

### 3.2. Models Evaluation

**Figure 2:**
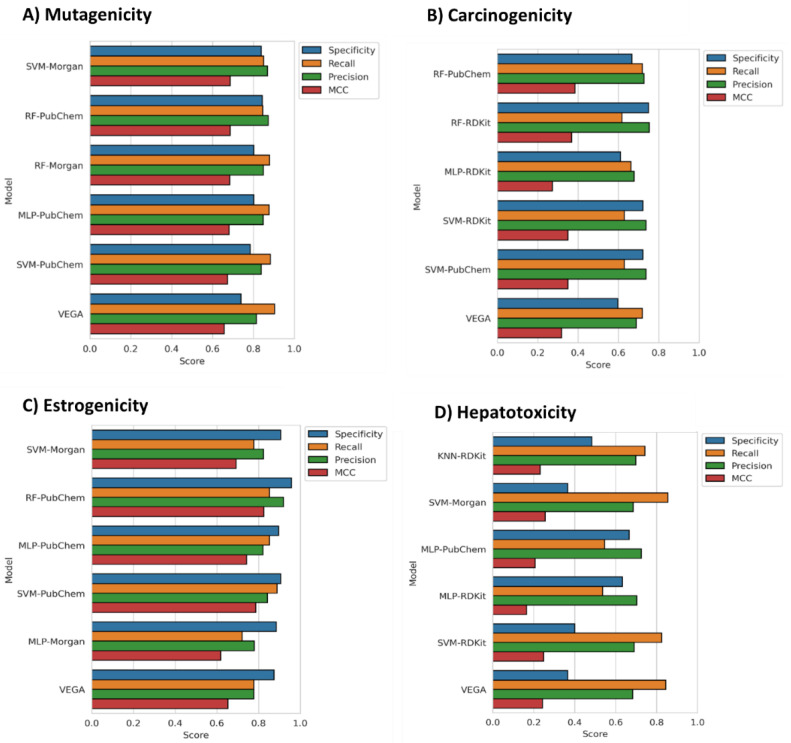
Performance evaluation results, based on test set prediction, obtained for the 5 top-scored models of the (**A**) mutagenicity, (**B**) carcinogenicity, (**C**) estrogenicity and (**D**) hepatotoxicity endpoint, in comparison with the reference models included in VEGA.

The five top-scored models of each endpoint were then subjected to a final evaluation consisting in predicting the potential toxicity of the test set molecules, which were not used for model training (external evaluation). The performance of the five models selected for each endpoint was evaluated in terms of Specificity, Recall and Precision, besides MCC (as reported in Materials and Methods). Figure 2 shows that our models obtained equal or better results than the reference models included in VEGA. Specifically, most of our models performed better than VEGA in terms of all metrics, with the exception of Recall, for which only a few models performed better than the reference ones. Moreover, although the SVM algorithm was the most present among the top-scored models of the different endpoints based on CV, RF was found to be the classifier that allowed to obtain the best results in most cases for the external evaluation.

The results obtained for the mutagenicity endpoint (Figure 2A), show that all of the selected models performed better than VEGA in terms of MCC. In particular, the model based on the RF algorithm combined with PubChem FPs, as well as that combining SVM with Morgan FPs, was found to be the best performing ones, performing equally and achieving the highest MCC, Precision and Specificity scores. Interestingly, the three remaining models, which achieved slightly lower MCC values, were found to perform better in Recall, with a score of 0.88. Nevertheless, all of our models were slightly inferior to VEGA in terms of Recall (0.90).

Concerning the carcinogenicity endpoint, all models except the MLP-based one outperformed VEGA in terms of MCC (Figure 2B). The model based on the RF algorithm trained with PubChem FPs showed the best performance and achieved an MCC value of 0.39 compared to the MCC of 0.32 shown by the reference model included in VEGA. Moreover, this was the only model equaling VEGA in terms of Recall. On the other hand, the model based on RF and RDKit FPs obtained the highest score for Precision and Specificity.

Even for the estrogenicity endpoint, four out of five models achieved higher MCC scores than that shown by the reference model in VEGA (Figure 2C). The trend shown in the results of the previous endpoints was also confirmed for the estrogenicity one, since RF and PubChem demonstrated to represent the best combination of classification algorithm and FPs, respectively. However, in this case, the RF-PubChem model showed not only the best MCC value (0.83), considerably higher than that obtained by VEGA (MCC = 0.65) but also a significant improvement in terms of Precision, Specificity and even Recall. Nevertheless, the model based on SVM and PubChem FPs was the model that achieved the best Recall value (0.89). Of note, this algorithm and FP combination showed the best results in terms of Recall also for the mutagenicity endpoint.

Consistent with the cross-validation results (Figure 1), hepatotoxicity was the endpoint for which the lowest predictive efficacies were generally obtained, although two out of five models outperformed the reference model belonging from VEGA in terms of MCC. Moreover, no RF-based model was included among the five top-scored ones; therefore, the trend observed for all other endpoints, showing the model based on RF algorithm and PubChem FPs as the best one, could not be confirmed in this case. Nevertheless, the best hepatotoxicity model was based on the combination of the SVM algorithm and Morgan FPs, which also showed the best results in the mutagenicity endpoint together with the RF-PubChem model. The SVM–Morgan model was found to be the best also in terms of Recall, performing better than VEGA. In terms of Precision and Specificity, the highest scores were achieved by the model based on MPL and PubChem FP. The low MCC values generally observed can be mainly rationalized by looking at the poor Specificity scores shown by most models, which are often below 0.5 as that reported for the reference model (Specificity = 0.37 for VEGA). On the contrary, for all models, high Precision scores were observed. These results highlight that all models performed better at correctly predicting hepatotoxic compounds than in identifying non-hepatotoxic molecules.

### 3.3. Consensus Strategy

In the attempt to improve the predictive performance achievable through the use of our models, we applied a consensus strategy that allowed us to combine the different predictions of the top-scored models selected for each endpoint. The reason behind using a consensus approach is based on the reliability it showed in improving the prediction of ligand dispositions in docking studies [30], proving to represent an effective and profitable approach for the successful identification of new hit compounds through virtual screening [31]. In this case, for applying a consensus strategy, we considered the probability scores (PSs) associated with the toxicity predictions provided by each selected model for each tested compound (see Materials and Methods for details). In order to obtain a merged collective prediction for each tested molecule, derived from the top-scored models of each endpoint, we calculated a consensus score (CS) by averaging the PSs associated with the toxicity predictions provided by each selected model. In particular, we calculated three different consensus predictions for each endpoint by combining the PSs of the top-3 (CS3), top-4 (CS4) and top-5 (CS5) models in terms of MCC obtained in the external evaluation (see also Tables S1–S4 in the Supplementary Materials). Hence, a compound was labeled as toxic only if the obtained CS was at least equal to or greater than 0.5. By following this approach, we aimed at enhancing the reliability of the toxicity predictions by reducing the potential bias generated by single models. In order to test the efficacy of the consensus approach, the same statistical parameters used for evaluating the performance of the single top-scored models (Specificity, Recall, Precision and MCC) were then calculated for the classification based on the three different CSs.

**Figure 3:**
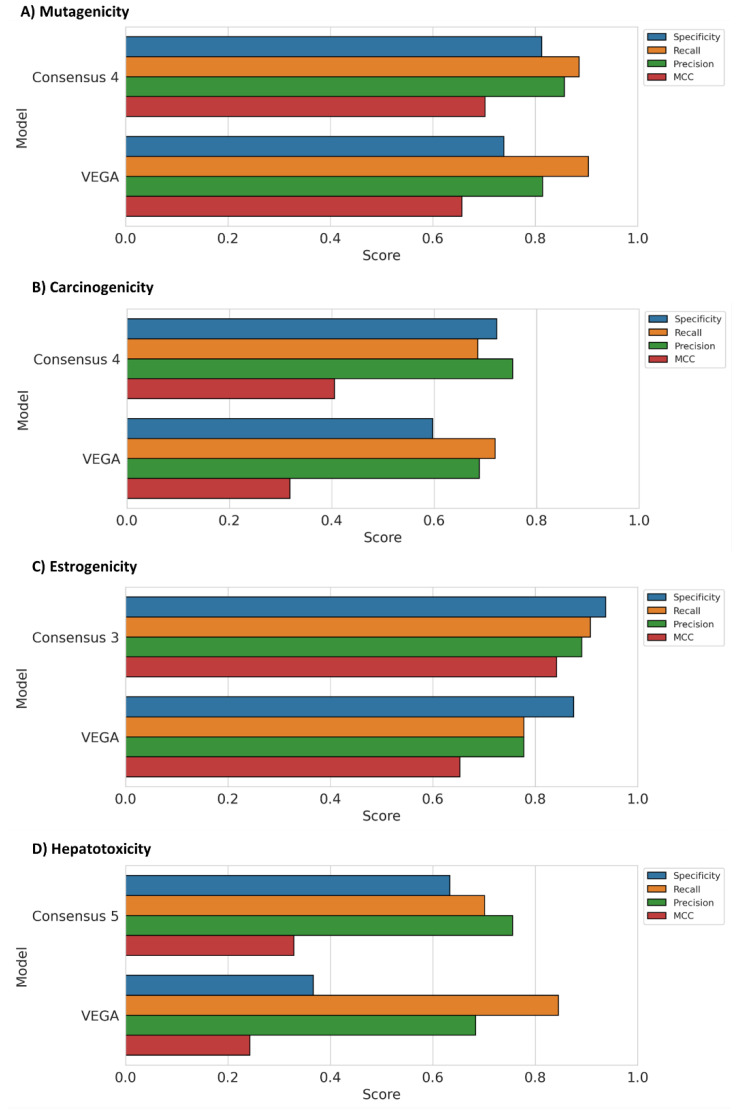
Statistical values obtained with the consensus strategy for the (**A**) mutagenicity, (**B**) carcinogenicity, (**C**) estrogenicity and (**D**) hepatotoxicity endpoints, compared with the reference models included in VEGA.

Figure 3 reports, for each endpoint, the results obtained for the consensus approach that showed the best performance, compared with the results obtained with the reference models included in VEGA. For the mutagenicity endpoint, consensus 3 and 4 were found to be the best strategies since they obtained an MCC of 0.72. On the other hand, consensus 5 also proved to be a valid approach, showing an MCC value (0.71) highly comparable with that of the other two consensus predictions (Table S1). Notably, consensus 3 and 4 outperformed the two best mutagenicity models, which showed an MCC of 0.69. Interestingly, consensus 4 achieved a lower Specificity than consensus 3 but showed a higher Recall value. Considering that both approaches outperformed the reference model in VEGA in terms of Specificity, we decided to select consensus 4 as the best strategy since it obtained the highest Recall score compared to both single models and the other consensus approaches. (Figure 3A).

Consensus 4 was also the best approach for the carcinogenicity endpoint, achieving an MCC of 0.41 compared to 0.39 obtained by the model based on the RF algorithm and PubChem FPs (Table S2). Consensus 4 performed better than the reference model included in VEGA in terms of MCC, Specificity and Precision (Figure 3B). On the contrary, consensus 3 and 5 showed a performance in terms of MCC (0.34) considerably lower than that observed for both consensus 4 and the best model (RF-PubChem).

The trend observed for the last two endpoints was not confirmed when analyzing the results of the estrogenicity endpoint, highlighting as the best approach consensus 3, which showed an MCC score of 0.84 and slightly outperformed both the best estrogenicity model (RF-PubChem) and the other two consensus approaches (Table S3). As shown in Figure 3C, in this case, we were able to obtain a strong performance improvement, with respect to the reference model belonging from VEGA (MCC = 0.65), in terms of all statistical metrics, including Recall, which reached a value of 0.91 versus 0.78 showed by VEGA.

Hepatotoxicity was the only endpoint where consensus 4 was the worst strategy. In this case, the largest improvement was found for consensus 5, thus considering the prediction probabilities of all five best models. Specifically, the MCC achieved by consensus 5 was 0.33, while a score of 0.26 was obtained with the best single hepatotoxicity model (SVM–Morgan, Table S4). The consensus 5 strategy outperformed the reference VEGA model for every metric except Recall (Figure 3D). In particular, the largest improvement was observed for Specificity (0.63), with an increase of 0.26 over VEGA (0.37); therefore, the consensus strategy was successful in strongly ameliorating the parameter that showed to have the most negative impact on the global performance of the single hepatotoxicity models.

### 3.4. Structure-Based Analysis of Toxicity Predictions

**Figure 4:**
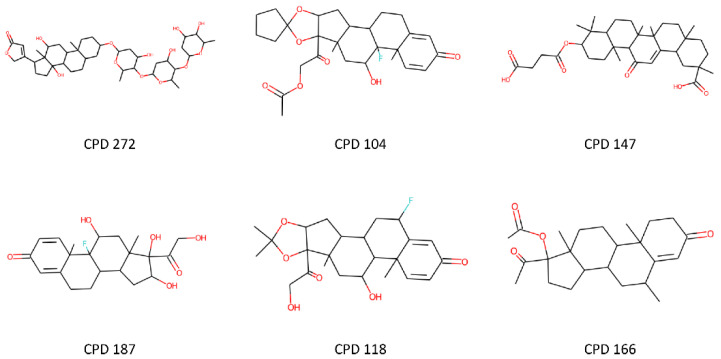
Non-hepatotoxic compounds sharing a steroid scaffold misclassified by the reference model from VEGA and correctly predicted by our consensus approach.

In order to obtain a deeper insight into the improvement in prediction reliability obtained using the consensus approach with respect to the single reference models included in VEGA, we analyzed the results of the test set predictions obtained for all four endpoints in order to check the structures of the compounds misclassified by VEGA and correctly classified by our strategy. The most interesting results were identified among the hepatotoxicity predictions. The reference hepatotoxicity model included in VEGA generated 60 false-positive predictions, i.e., non-hepatotoxic compounds that were predicted as toxic, while 38 of these compounds (63%) were correctly classified by our consensus strategy combining the top 5 models herein developed (see Section 3.3). Interestingly, a cluster of 6 compounds sharing a common steroid structure was identified among these 38 molecules (Figure 4).

The hepatotoxicity training set was then subjected to a structural analysis aimed at identifying compounds chemically similar to this cluster of molecules. The results showed a cluster of 43 structurally related molecules with a similar steroid or steroid-like scaffold. Of note, 21 (49%) of these were labeled as non-hepatotoxic, whereas the remaining 22 (51%) were labeled as hepatotoxic. This situation, in which structurally similar training set compounds are evenly labeled as toxic and non-toxic, represents a big challenge for any ML model, which needs to derive subtle feature patterns able to discriminate toxic from non-toxic molecules properly.

The fact that our consensus approach succeeded at correctly predicting the six structurally related compounds (Figure 4) highlights the power of the consensus strategy, which exploits the advantage of combining models based on different molecular representations and classification algorithms that can thus extrapolate multiple types of hidden patterns allowing the proper classification of the compounds. On the other hand, the use of a consensus approach makes it difficult to identify common structural motifs linked to toxicity, as well as to derive structure–activity relationship (SAR) data, due to the fact that different models decipher and interpret molecular structures and features in a different way. If this represents an advantage in terms of prediction reliability, it constitutes a disadvantage in terms of interpretation of predictions. Nevertheless, SAR data and toxicophores may be extrapolated using simple fragment-based approaches that are independent of the machine learning algorithms used for toxicity prediction. For instance, by analyzing the frequency with which PubChem substructures are found among toxic and non-toxic compounds within the estrogenicity training set, we can find that the frequency of a specific fragment (including two oxygen atoms connected to two vicinal aromatic carbons) in toxic molecules (12%) is 8-fold higher than in non-toxic compounds (1.4%), thus suggesting such substructure as a potential estrogenic toxicophore (see also Figures S1 and S2 in the Supplementary Materials). Although this is out of the scope of the present work, which is focused on maximizing the toxicity prediction reliability, implementation of such approaches will be the target of future studies, with the aim of including this aspect in our models.

### 3.5. VenomPred Web Tool

**Figure 5:**
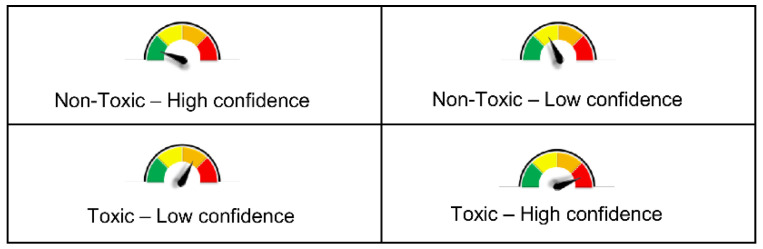
The graphical representation provided by VenomPred related to the confidence level of each prediction, derived from the *Probability* value.

With the aim of creating a freely accessible platform for multiple toxicity predictions, we developed the web tool VenomPred, available on the following page of our research group’s website http://www.mmvsl.it/wp/venompred/. VenomPred allows toxicological predictions of potential mutagenicity, carcinogenicity, estrogenicity and hepatotoxicity of small molecules employing the best performing models herein developed for each endpoint by following the consensus approach. In particular, for each endpoint, the consensus strategy that showed the best results was implemented in our web tool. VenomPred presents an intuitive and user-friendly interface. The users only need to provide the SMILES (Simplified Molecular-Input Line-Entry System) strings of the compounds to be evaluated, which can be obtained by simply drawing the molecular structures on the integrated 2D sketcher [32]. In order to complete the request, it is necessary to select at least one of the four endpoints available; after the request is submitted, the web tool computes the consensus toxicity prediction for each endpoint. A report providing the results for each analyzed molecule is then sent by email. The report includes a “*Probability*” value related to each toxicity prediction requested, which corresponds to the value of the consensus score expressed as a percentage. In order to facilitate the interpretation of results, VenomPred report also includes a graphical representation related to the confidence level of each prediction, derived from the *Probability* value, which is divided into four different ranges: (*a*) *Probability* < 25%, (*b*) 25% ≤ *Probability* < 50%, (*c*) 50% ≤ *Probability* < 75% and (*d*) 75% ≤ *Probability*. The first two ranges, *a* and *b*, respectively, indicate a high and low confidence level in the prediction of a molecule as harmless, while ranges *c* and *d* indicate a low and high level of confidence, respectively, for a prediction of toxicity. The four different *Probability* ranges *a*, *b*, *c* and *d* were then, respectively, color-coded as green, yellow, orange and red sections of the graphical indicator associated with each prediction within the VenomPred report (Figure 5).

Figure S3 in the Supplementary Materials shows a typical report including predictions for all available endpoints related to a single compound. In this case, the query compound is Nithiazide (CAS number: 139-94-6), a veterinary medicine used in the past as an antiprotozoal agent whose mutagenicity and carcinogenicity are reported in the literature [33]. VenomPred platform confirms the toxicity of the query compound by correctly predicting it as mutagenic and carcinogenic with high confidence (*Probability* of 89% and 78% for mutagenicity and carcinogenicity, respectively), while a *Probability* of potential hepatotoxic and estrogenic effect of 37% and 7%, respectively, is estimated, and thus the compound is predicted as safe with low and high confidence, respectively.

## 4. Conclusions

A rapid and efficient assessment of the potential toxicity of small molecules is a hot topic for the whole scientific community and represents a field of particular interest in drug discovery since the early evaluation of the toxicity profile of a lead compound is of great importance for its development. In this context, we developed machine learning (ML) models allowing the evaluation of the potential mutagenicity, carcinogenicity, estrogenicity and hepatotoxicity of small molecules due to the great impact of these four toxicological aspects in drug discovery and development. All models were trained and evaluated using the training and test sets of reference models included in the VEGA QSAR platform. When compared to VEGA, our models were often able to achieve better results in terms of multiple statistical parameters employed in performance evaluations, such as Matthew’s Correction Coefficient. In order to further improve the prediction reliability, we applied three different consensus approaches that combined the predictions of the three, four and five best models generated for each endpoint. The consensus approach was able to outperform both the reference models included in VEGA and the best single models developed for each endpoint. The best consensus strategy for each endpoint was thus implemented in the VenomPred platform (http://www.mmvsl.it/wp/venompred/), a freely available web tool easily accessible even to non-expert users. The workflow used for the generation of the ML models herein reported may be thus profitably applied for the development of new efficient models focused on other toxicity endpoint predictions that can be implemented in the VenomPred platform.

## Figures and Tables

**Table 1:** Total number of molecules present in each training and test set, including their classification according to experimental value, was employed for model building and evaluation.

**Mutagenicity Model**			
**Set**	**Total**	**Mutagen**	**Non-mutagen**
Training	3367	1883	1484
Test	798	446	352
**Carcinogenicity Model**			
**Set**	**Total**	**Carcinogenic**	**Non-carcinogenic**
Training	645	333	312
Test	161	89	72
**Estrogenicity Model**			
**Set**	**Total**	**Active**	**Inactive**
Training	656	234	422
Test	150	54	96
**Hepatotoxicity Model**			
**Set**	**Total**	**Toxic**	**Non-toxic**
Training	760	408	352
Test	157	97	60

## Data Availability

http://www.mmvsl.it/wp/venompred/.

## Supplementary Materials

The following supporting information can be downloaded at: https://www.mdpi.com/article/10.3390/ijms23042105/s1.
