# Supplementary material for: VenomPred: A Machine Learning Based Platform for Molecular Toxicity Predictions
Source: Int J Mol Sci. 2022 Feb 14;23(4):2105. doi: 10.3390/ijms23042105 (PMC8877213; doi:10.3390/ijms23042105)
Supplement: Supplementary file 1 [file ijms-23-02105-s001.zip › ijms-1568718-supplementary.pdf]

International Journal of *Molecular Sciences*

MDPI

Supporting Information

VenomPred: A Machine Learning Based Platform for Molecular Toxicity Predictions

Salvatore Galati [1,†], Miriana Di Stefano [1,2,†], Elisa Martinelli [1], Marco Macchia [1], Adriano Martinelli [1], Giulio Poli [1,*] and Tiziano Tuccinardi [1,3]

[1] Department of Pharmacy, University of Pisa, 56126 Pisa, Italy; salvatore.galati@phd.unipi.it (S.G.); miriana.distefano@phd.unipi.it (M.D.S.); e.martinelli3@studenti.unipi.it (E.M.); marco.macchia@unipi.it (M.M.); marti@adrianomartinelli.it (A.M.); tiziano.tuccinardi@unipi.it (T.T.)
[2] Department of Life Sciences, University of Siena, 53100 Siena, Italy
[3] Center for Biotechnology, Sbarro Institute for Cancer Research and Molecular Medicine, College of Science and Technology, Temple University, Philadelphia, PA 19122, USA
* Correspondence: giulio.poli@unipi.it; Tel.: +39-050-2219603
† These authors contributed equally to this work.

Table of Contents

Table S1. Evaluation results based on test set prediction for the mutagenicity endpoint. Page S2

Table S2. Evaluation results based on test set prediction for the carcinogenicity endpoint. Page S2

Table S3. Evaluation results based on test set prediction for the estrogenicity endpoint. Page S2

Table S4. Evaluation results based on test set prediction for the hepatotoxicity endpoint. Page S3

Figure S1. Estrogenic training set compounds showing a potential toxicophore. Page S3

Figure S2. Non-estrogenic training set compounds showing a potential toxicophore. Page S4

Figure S3. Example of toxicity prediction report produced by VenomPred web tool. Page S4

Table S1. Performance evaluation results, based on test set prediction, obtained with the 5 top-scored models and consensus strategy compared to reference models included in VEGA for the mutagenicity endpoint.

Model	Specificity	Recall	Precision	MCC
RF-PubChem	0.84	0.85	0.87	0.69
SVM-Morgan	0.84	0.85	0.87	0.69
RF-Morgan	0.80	0.88	0.85	0.68
MLP-PubChem	0.80	0.88	0.85	0.68
SVM-PubChem	0.78	0.88	0.84	0.67
Consensus 3	0.84	0.88	0.87	0.72
Consensus 4	0.83	0.89	0.87	0.72
Consensus 5	0.83	0.88	0.87	0.71
VEGA	0.74	0.90	0.81	0.66

Table S2. Performance evaluation results, based on test set prediction, obtained with the 5 top-scored models and consensus strategy compared to reference models included in VEGA for the carcinogenicity endpoint.

Model	Specificity	Recall	Precision	MCC
RF-PubChem	0.67	0.72	0.73	0.39
RF-RDKit	0.75	0.62	0.75	0.37
SVM-RDKit	0.72	0.63	0.74	0.35
SVM-PubChem	0.72	0.63	0.74	0.35
MLP-RDKit	0.61	0.66	0.68	0.27
Consensus 3	0.69	0.65	0.73	0.34
Consensus 4	0.72	0.69	0.75	0.41
Consensus 5	0.68	0.66	0.72	0.34
VEGA	0.60	0.72	0.69	0.32

Table S3. Performance evaluation results, based on test set prediction, obtained with the 5 top-scored models and consensus strategy compared to reference models included in VEGA for the estrogenicity endpoint.

Model	Specificity	Recall	Precision	MCC
RF-PubChem	0.96	0.85	0.92	0.83
SVM-PubChem	0.91	0.89	0.84	0.79
MLP-PubChem	0.90	0.85	0.82	0.74
SVM-Morgan	0.91	0.78	0.82	0.69
MLP-Morgan	0.89	0.72	0.78	0.62
Consensus 3	0.94	0.91	0.89	0.84
Consensus 4	0.94	0.89	0.89	0.83
Consensus 5	0.95	0.85	0.90	0.81
VEGA	0.88	0.78	0.78	0.65

Table S4. Performance evaluation results, based on test set prediction, obtained with the 5 top-scored models and consensus strategy compared to reference models included in VEGA for the hepatotoxicity endpoint.

Model	Specificity	Recall	Precision	MCC
SVM-Morgan	0.37	0.86	0.69	0.26
SVM-RDKit	0.40	0.83	0.69	0.25
KNN-RDKit	0.48	0.74	0.70	0.23
MLP-PubChem	0.67	0.55	0.73	0.21
MLP-RDKit	0.63	0.54	0.70	0.17
Consensus 3	0.42	0.85	0.70	0.29
Consensus 4	0.58	0.69	0.73	0.27
Consensus 5	0.63	0.70	0.76	0.33
VEGA	0.37	0.85	0.68	0.24

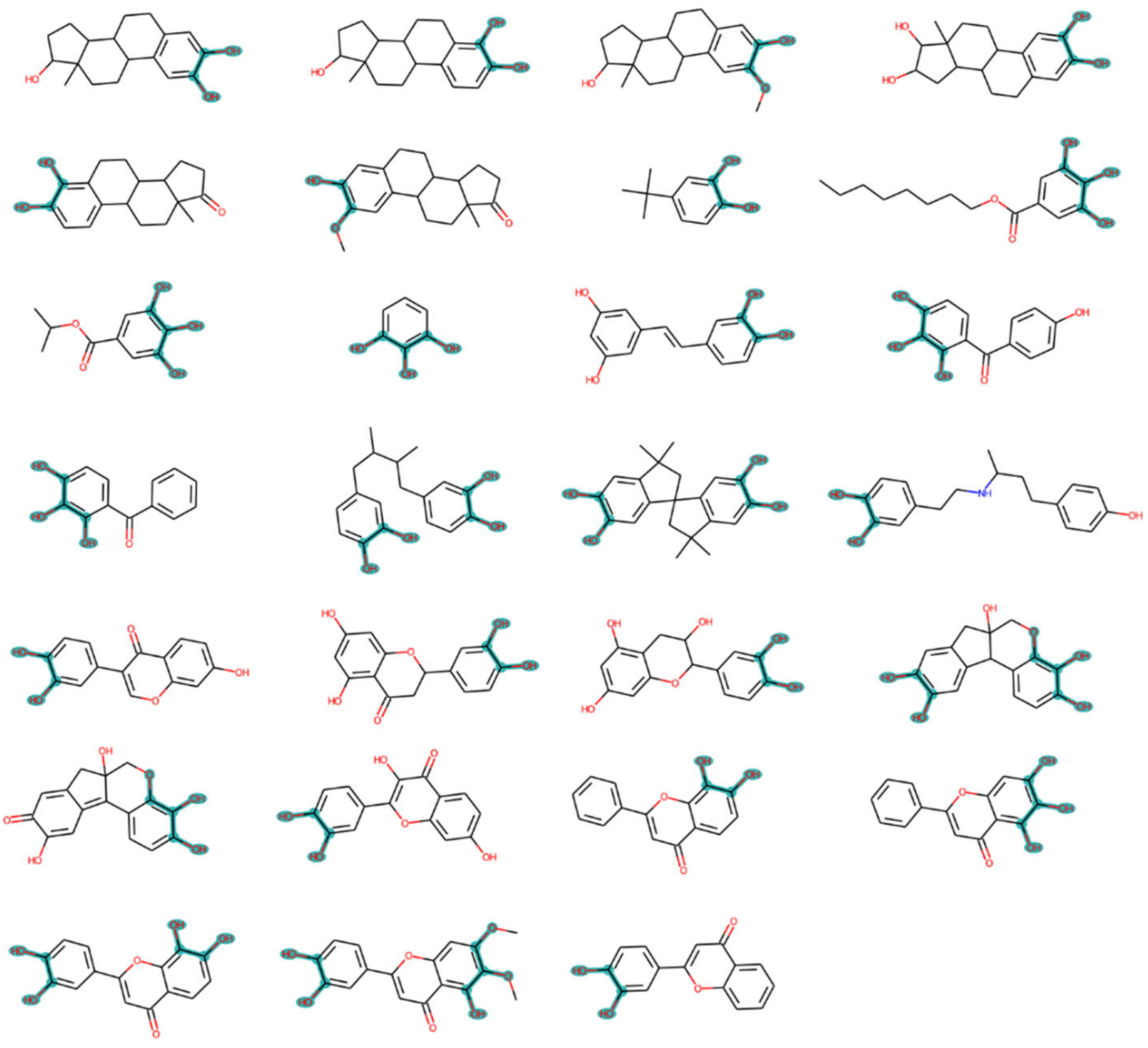

Figure S1. Compounds endowed with estrogenic activity included in the estrogenicity training set showing the potential estrogenic toxicophore (a fragment including two oxygen atoms connected to two vicinal aromatic carbons), highlighted in cyan. In some of the compound, the fragment is observed more than once.

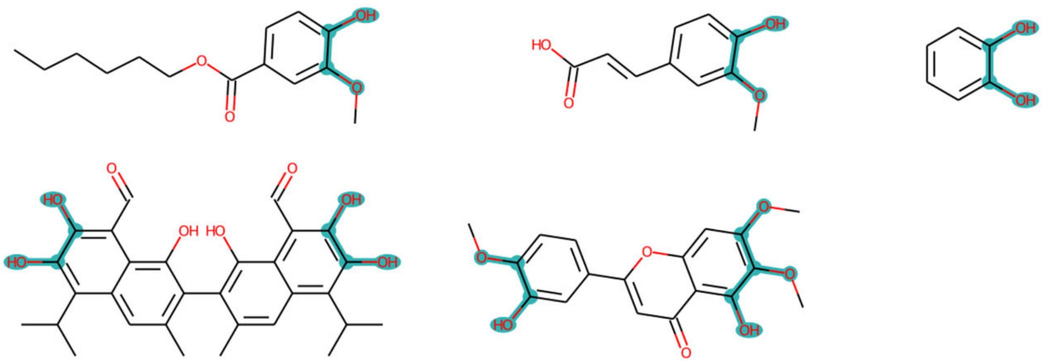

Figure S2. Compounds devoid of estrogenic activity included in the estrogenicity training set showing the potential estrogenic toxicophore (a fragment including two oxygen atoms connected to two vicinal aromatic carbons), highlighted in cyan. In some of the compound, the fragment is observed more than once.

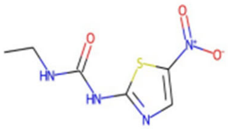

**Name**	Entry_1
**SMILES**	CCNC(=O)NC1=NC=C(S1)[N+](=O)[O-]

Endpoint	Probability
Mutagenicity	89 % 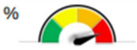
Carcinogenicity	78 % 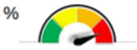

Endpoint	Probability
Hepatotoxicity	37 % 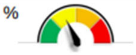
Estrogenicity	7 % 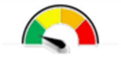

Figure S3. Example of toxicity prediction report produced by VenomPred web tool. The query compound is Nithiazide (CAS# 139-94-6).
